# Good-for-MDPs Automata for Probabilistic Analysis and Reinforcement Learning

**DOI:** 10.1007/978-3-030-45190-5_17

**Published:** 2020-03-13

**Authors:** Ernst Moritz Hahn, Mateo Perez, Sven Schewe, Fabio Somenzi, Ashutosh Trivedi, Dominik Wojtczak

**Affiliations:** 8 Johannes Kepler University, Linz, Austria; 9 University of Birmingham, Birmingham, UK; 10 School of EEECS, Queen’s University Belfast, Belfast, UK; 11 State Key Laboratory of Computer Science, Institute of Software, CAS, Beijing, People’s Republic of China; 12 University of Colorado Boulder, Boulder, USA; 13 University of Liverpool, Liverpool, UK

## Abstract

We characterize the class of nondeterministic $\omega$-automata that can be used for the analysis of finite Markov decision processes (MDPs). We call these automata ‘good-for-MDPs’ (GFM). We show that GFM automata are closed under classic simulation as well as under more powerful simulation relations that leverage properties of optimal control strategies for MDPs. This closure enables us to exploit state-space reduction techniques, such as those based on direct and delayed simulation, that guarantee simulation equivalence. We demonstrate the promise of GFM automata by defining a new class of automata with favorable properties—they are Büchi automata with low branching degree obtained through a simple construction—and show that going beyond limit-deterministic automata may significantly benefit reinforcement learning.

### 1 Introduction

System specifications are often captured in the form of finite automata over infinite words ($\omega$-automata), which are then used for model checking, synthesis, and learning. Of the commonly-used types of $\omega$-automata, Büchi automata have the simplest acceptance condition, but require nondeterminism to recognize all $\omega$-regular languages. Nondeterministic machines can use unbounded look-ahead to resolve nondeterministic choices. However, important applications—like reactive synthesis or model checking and reinforcement learning (RL) for Markov Decision Process (MDPs [23])—have a game setting, which restrict the resolution of nondeterminism to be based on the past.

Being forced to resolve nondeterminism on the fly, an automaton may end up rejecting words it should accept, so that using it can lead to incorrect results. Due to this difficulty, initial solutions to these problems have been based on deterministic automata—usually with Rabin or parity acceptance conditions. For two-player games, Henzinger and Piterman proposed the notion of *good-for-games (GFG)* automata [15]. These are nondeterministic automata that simulate [21,14,9] a deterministic automaton that recognizes the same language. The existence of a simulation strategy means that nondeterministic choices can be resolved without look-ahead.

* This work has been supported by the National Natural Science Foundation of China (Grant Nr. 61532019), EPSRC grants EP/M027287/1 and EP/P020909/1, and a CU Boulder RIO grant.

The situation is better in the case of probabilistic model checking, because the game for which a strategy is sought is played on an MDP against "blind nature," rather than against a strategic opponent who may take advantage of the automaton's inability to resolve nondeterminism on the fly. As early as 1985, Vardi noted that probabilistic model checking can be performed with Büchi automata endowed with a limited form of nondeterminism [34]. *Limit deterministic Büchi automata (LDBA)* [4,11,29] perform no nondeterministic choice after seeing an accepting transition. Still, they recognize all $\omega$-regular languages and are, under mild restrictions [29], *suitable* for probabilistic model checking.

**Related Work.** The production of deterministic and limit deterministic automata for model checking has been intensively studied [24,22,1,26,33,32,27,29,8,30,20], and several tools are available to produce different types of automata, incl. MoChiBA/Owl [29,30,20], LTL3BA [1], GOAL [33,32], SPOT [8], Rabinizer [19], and Büchifier [16].

So far, only deterministic and a (slightly restricted [29]) class of limit deterministic automata have been considered for probabilistic model checking [34,4,11,29]. Thus, while there have been advances in the efficient production of such automata [11,29,30,20], the consideration of suitable LDBAs by Courcoubetis and Yannakakis in 1988 [3] has been the last time when a fundamental change in the automata foundation of MDP model checking has occurred.

**Contribution.** The simple but effective observation that simulation preserves the suitability for MDPs (for both traditional simulation and the AEC simulation we introduce) extends the class of automata that can be used in the analysis of MDPs. This provides us with three advantages: The first advantage is that we can now use a wealth of simulation based statespace reduction techniques [7,31,10,9] on an automaton $\mathcal{A}$ (e.g. an SLDBA) that we would otherwise use for MDP model checking. The second advantage is that we can use $\mathcal{A}$ to check if a different language equivalent automaton, such as an NBA $\mathcal{B}$ (e.g. an NBA from which $\mathcal{A}$ is derived) simulates $\mathcal{A}$. For this second advantage, we can dip into the more powerful class of AEC simulation we define in Section 4 that use properties of winning strategies on finite MDPs. While this is not a complete method for identifying GFM automata, our experimental results indicate that the GFM property is quite frequent for NBAs constructed from random formulas, and can often be established efficiently, while providing a significant statespace reduction and thus offering a significant advantage for model checking.

A third advantage is that we can use the additional flexibility to tailor automata for different applications than model checking, for which specialized automata classes have not yet been developed. We demonstrate this for model-free reinforcement learning (RL). We argue that RL benefits from three properties that are less important in model checking: The first—easy to measure—property is a small number of successors, the second and third, are *cautiousness*, the scope for making wrong decisions, and *forgiveness*, the resilience against making wrong decisions, respectively.

A small number of successors is a simple and natural goal for RL, as the lack of an explicit model means that the product space of a model and an automaton cannot be evaluated backwards. In a forward analysis, it matters that nondeterministic choices have to be modeled by enriching the decisions in the MDPs with the choices made by the automaton. For LDBAs constructed from NBAs, this means guessing a suitable subset of the reachable states when progressing to the deterministic part of the automaton, meaning a number of choices that is exponential in the NBA. We show that we can instead use *slim automata* in Section 3.2 as a first example of NBAs that are good-for-MDPs, but not limit deterministic. They have the appealing property that their branching degree is at most two, while keeping the Büchi acceptance mechanism that works well with RL [12]. (Slim automata can also be used for model checking, but they don't provide similar advantages over suitable LDBAs there, because the backwards analysis used in model checking makes selecting the correct successor trivial.)

Cautiousness and forgiveness are further properties, which are—while harder to quantify—very desirable for RL: LDBAs, for example, suffer from having to make a *correct* choice when moving into the deterministic part of the automaton, and they have to make this correct choice from a very large set of nondeterministic transitions. While this is unproblematic for standard model checking algorithms that are based on backwards analysis, applications like RL that rely on forward analysis can be badly affected when more (wrong) choices are offered, and when wrong choices cannot be rectified. Cautiousness and forgiveness are a references to this: an automaton is more *cautious* if it has less scope for making wrong decisions and more *forgiving* if it allows for correcting previously made decisions (cf. Figure 5 for an example). Our experiments (cf. Section 5) indicate that cautiousness and forgiveness are beneficial for RL.

**Organization of the Paper.** After the preliminaries, we introduce the "good-for-MDP" property (Section 3) and show that it is preserved by simulation, which enables all minimization techniques that offer the simulation property (Section 3.1). In Section 3.2 we use this observation to construct slim automata—NBAs with a branching degree of 2 that are neither limit deterministic nor good-for-games—as an example of a class of automata that becomes available for MDP model checking and RL. We then introduce a more powerful simulation relation, AEC simulation, that suffices to establish that an automaton is good-for-MDPs (Section 4). In Section 5, we evaluate the impact of the contributions of the paper on model checking and reinforcement learning algorithms.

### 2 Preliminaries

A *nondeterministic Büchi automaton* is a tuple $\mathcal{A} = \langle \Sigma, Q, q_0, \Delta, \Gamma \rangle$, where $\Sigma$ is a finite *alphabet*, $Q$ is a finite set of *states*, $q_0 \in Q$ is the *initial state*, $\Delta \subseteq Q \times \Sigma \times Q$ are transitions, and $\Gamma \subseteq Q \times \Sigma \times Q$ is the transition-based *acceptance condition*.

A *run* $r$ of $\mathcal{A}$ on $w \in \Sigma^\omega$ is an $\omega$-word $r_0, w_0, r_1, w_1, \ldots$ in $(Q \times \Sigma)^\omega$ such that $r_0 = q_0$ and, for $i > 0$, it is $(r_{i-1}, w_{i-1}, r_i) \in \Delta$. We write $\inf(r)$ for the set of transitions that appear infinitely often in the run $r$. A run $r$ of $\mathcal{A}$ is *accepting* if $\inf(r) \cap \Gamma \neq \emptyset$.

The *language*, $L_\mathcal{A}$, of $\mathcal{A}$ (or, *recognized* by $\mathcal{A}$) is the subset of words in $\Sigma^\omega$ that have accepting runs in $\mathcal{A}$. A language is $\omega$-*regular* if it is accepted by a Büchi automaton. An automaton $\mathcal{A} = \langle \Sigma, Q, Q_0, \Delta, \Gamma \rangle$ is *deterministic* if $(q, \sigma, q'), (q, \sigma, q'') \in \Delta$ implies $q' = q''$. $\mathcal{A}$ is *complete* if, for all $\sigma \in \Sigma$ and all $q \in Q$, there is a transition $(q, \sigma, q') \in \Delta$. A word in $\Sigma^\omega$ has exactly one run in a deterministic, complete automaton.

A *Markov decision process (MDP)* $\mathcal{M}$ is a tuple $(S, A, T, \Sigma, L)$ where $S$ is a finite set of states, $A$ is a finite set of *actions*, $T : S \times A \rightharpoondown \mathcal{D}(S)$, where $\mathcal{D}(S)$ is the set of probability distributions over $S$, is the *probabilistic transition (partial) function*, $\Sigma$ is an alphabet, and $L : S \times A \times S \to \Sigma$ is the *labeling function* of the set of transitions. For a state $s \in S$, $A(s)$ denotes the set of actions available in $s$. For states $s, s' \in S$ and $a \in A(s)$, we have that $T(s,a)(s')$ equals $\Pr(s'|s,a)$.

A *run* of $\mathcal{M}$ is an $\omega$-word $s_0, a_1, \ldots \in S \times (A \times S)^\omega$ such that $\Pr(s_{i+1}|s_i, a_{i+1}) > 0$ for all $i \geq 0$. A finite run is a finite such sequence. For a *run* $r = s_0, a_1, s_1, \ldots$ we define the corresponding labeled run as $L(r) = L(s_0, a_1, s_1), L(s_1, a_2, s_2), \ldots \in \Sigma^\omega$. We write $\Omega(\mathcal{M})$ ($\mathrm{Paths}(\mathcal{M})$) for the set of runs (finite runs) of $\mathcal{M}$ and $\Omega_s(\mathcal{M})$ ($\mathrm{Paths}_s(\mathcal{M})$) for the set of runs (finite runs) of $\mathcal{M}$ starting from state $s$. When the MDP is clear from the context we drop the argument $\mathcal{M}$.

A strategy in $\mathcal{M}$ is a function $\mu : \mathrm{Paths} \to \mathcal{D}(A)$ such that $\mathrm{supp}(\mu(r)) \subseteq A(\mathrm{last}(r))$, where $\mathrm{supp}(d)$ is the support of $d$ and $\mathrm{last}(r)$ is the last state of $r$. Let $\Omega_\mu^{\mathcal{M}}(s)$ denote the subset of runs $\Omega^{\mathcal{M}}(s)$ that correspond to strategy $\mu$ and initial state $s$. Let $\Pi_{\mathcal{M}}$ be the set of all strategies. We say that a strategy $\mu$ is *pure* if $\mu(r)$ is a point distribution for all runs $r \in \mathrm{Paths}$ and we say that $\mu$ is *positional* if $\mathrm{last}(r) = \mathrm{last}(r')$ implies $\mu(r) = \mu(r')$ for all runs $r, r' \in \mathrm{Paths}$. The behavior of an MDP $\mathcal{M}$ under a strategy $\mu$ with starting state $s$ is defined on a probability space $(\Omega_s^\mu, \mathcal{F}_s^\mu, \Pr_s^\mu)$ over the set of infinite runs of $\mu$ from $s$.

### 3 Good-for-MDP (GFM) Automata

Given an MDP $\mathcal{M}$ and an automaton $\mathcal{A} = \langle \Sigma, Q, q_0, \Delta, \Gamma \rangle$, we want to compute an optimal strategy satisfying the objective that the run of $\mathcal{M}$ is in the language of $\mathcal{A}$. We define the semantic satisfaction probability for $\mathcal{A}$ and a strategy $\mu$ from state $s$ as:

$$\mathsf{PSem}_{\mathcal{A}}^{\mathcal{M}}(s,\mu) = \Pr{}_s^\mu\{r \in \Omega_s^\mu : L(r) \in L_{\mathcal{A}}\} \text{ and } \mathsf{PSem}_{\mathcal{A}}^{\mathcal{M}}(s) = \sup_{\mu \in \Pi_{\mathcal{M}}} \left(\mathsf{PSem}_{\mathcal{A}}^{\mathcal{M}}(s,\mu)\right).$$

When using automata for the analysis of MDPs, we need a syntactic variant of the acceptance condition. Given an MDP $\mathcal{M} = (S, A, T, \Sigma, L)$ with initial state $s_0 \in S$ and automaton $\mathcal{A} = \langle \Sigma, Q, q_0, \Delta, \Gamma \rangle$, the *product* $\mathcal{M} \times \mathcal{A} = (S \times Q, (s_0, q_0), A \times Q, T^\times, \Gamma^\times)$ is an MDP [17] augmented with an initial state $(s_0, q_0)$ and accepting transitions $\Gamma^\times$. The (partial) function $T^\times : (S \times Q) \times (A \times Q) \rightharpoonup \mathcal{D}(S \times Q)$ is defined by

$$T^\times((s,q),(a,q'))((s',q')) = \begin{cases} T(s,a)(s') & \text{if } (q, L(s,a,s'), q') \in \Delta \\ \text{undefined} & \text{otherwise.} \end{cases}$$

Finally, $\Gamma^\times \subseteq (S \times Q) \times (A \times Q) \times (S \times Q)$ is defined by $((s,q),(a,q'),(s',q')) \in \Gamma^\times$ if, and only if, $(q, L(s,a,s'), q') \in \Gamma$ and $T(s,a)(s') > 0$. A strategy $\mu$ on the MDP defines a strategy $\mu^\times$ on the product, and vice versa. We define the syntactic satisfaction probabilities as

$$\mathsf{PSyn}_{\mathcal{A}}^{\mathcal{M}}((s,q),\mu^\times) = \Pr{}_s^\mu\{r \in \Omega_{(s,q)}^{\mu^\times}(\mathcal{M} \times \mathcal{A}) : \inf(r) \cap \Gamma^\times \neq \emptyset\}, \quad \text{and}$$

$$\mathsf{PSyn}_{\mathcal{A}}^{\mathcal{M}}(s) = \sup_{\mu^\times \in \Pi_{\mathcal{M} \times \mathcal{A}}} \left(\mathsf{PSyn}_{\mathcal{A}}^{\mathcal{M}}((s,q_0),\mu^\times)\right).$$

Note that $\mathsf{PSyn}_{\mathcal{A}}^{\mathcal{M}}(s) = \mathsf{PSem}_{\mathcal{A}}^{\mathcal{M}}(s)$ holds for a deterministic $\mathcal{A}$. In general, $\mathsf{PSyn}_{\mathcal{A}}^{\mathcal{M}}(s) \leq \mathsf{PSem}_{\mathcal{A}}^{\mathcal{M}}(s)$ holds, but equality is not guaranteed because the optimal resolution of nondeterministic choices may require access to future events (see Figure 1).

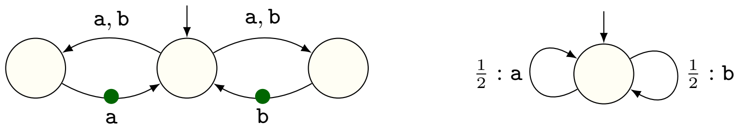

**Fig. 1.** An NBA, which accepts all words over the alphabet $\{a, b\}$, that is not good for MDPs. The dotted transitions are accepting. For the Markov chain on the right where the probability of $a$ and $b$ is $\frac{1}{2}$, the chance that the automaton makes infinitely many correct predictions is 0

**Definition 1 (GFM automata).** *An automaton $\mathcal{A}$ is* good for MDPs *if, for all MDPs $\mathcal{M}$, $\mathsf{PSyn}^{\mathcal{M}}_{\mathcal{A}}(s_0) = \mathsf{PSem}^{\mathcal{M}}_{\mathcal{A}}(s_0)$ holds, where $s_0$ is the initial state of $\mathcal{M}$.*

For an automaton to match $\mathsf{PSem}^{\mathcal{M}}_{\mathcal{A}}(s_0)$, its nondeterminism is restricted not to rely heavily on the future; rather, it must possible to resolve the nondeterminism on-the-fly. For example, the Büchi automaton presented on the left of Figure 1, which has to guess whether the next symbol is a or b, is not good for MDPs, because the simple Markov chain on the right of Figure 1 does not allow resolution of its nondeterminism on-the-fly.

There are three families of automata that are known to be good for MDPs: (1) deterministic automata, (2) good for games automata [15,18], and (3) limit deterministic automata that satisfy a few side constraints [4,11,29].

A *limit-deterministic* Büchi automaton (LDBA) is a nondeterministic Büchi automaton (NBA) $\mathcal{A} = \langle \Sigma, Q_i \cup Q_f, q_0, \Delta, \Gamma \rangle$ such that $Q_i \cap Q_f = \emptyset$; $q_0 \in Q_i$; $\Gamma \subseteq Q_f \times \Sigma \times Q_f$; $(q, \sigma, q'), (q, \sigma, q'') \in \Delta$ and $q, q' \in Q_f$ implies $q' = q''$; and $(q, \sigma, q') \in \Delta$ and $q \in Q_f$ implies $q' \in Q_f$. An LDBA behaves deterministically once it has seen an accepting transition. Usual LDBA constructions [11,29] produce GFM automata. We refer to LDBAs with this property as *suitable* (SLDBAs), cf. Theorem 1.

In the context of RL, techniques based on SLDBAs are particularly useful, because these automata use the Büchi acceptance condition, which can be translated to reachability goals. Good for games and deterministic automata require more complex acceptance conditions, like parity, that do not have a natural translation into rewards [12].

Using SLDBA [4,11,29] has the drawback that they naturally have a high branching degree in the initial part, as they naturally allow for many different transitions to the accepting part of the LDBA. This can be avoided, but to the cost of a blow-up and a more complex construction and data structure [29]. We therefore propose an automata construction that produces NBAs with a small branching degree—it never produces more than two successors. We call these automata *slim*. The resulting automata are not (normally) limit deterministic, but we show that they are good for MDPs.

Due to technical dependencies we start with presenting a second observation, namely that automata that *simulate* language equivalent GFM automata are GFM. As a side result, we observe that the same holds for good-for-games automata. The side result is not surprising, as good-for-games automata were defined through simulation of deterministic automata [15]. But, to the best of our knowledge, the observation from Corollary 1 has not been made yet for good-for-games automata.

#### 3.1 Simulating GFM

An automaton $\mathcal{A}$ *simulates* an automaton $\mathcal{B}$ if the duplicator wins the *simulation game*. The simulation game is played between a duplicator and a spoiler, who each control a pebble, which they move along the edges of $\mathcal{A}$ and $\mathcal{B}$, respectively. The game is started by the spoiler, who places her pebble on an initial state of $\mathcal{B}$. Next, the duplicator puts his pebble on an initial state of $\mathcal{A}$. The two players then take turns, always starting with the spoiler choosing an input letter and a transition for that letter in $\mathcal{B}$, followed by the duplicator choosing a transition for the same letter in $\mathcal{A}$. This way, both players produce an infinite run of their respective automaton. The duplicator has two ways to win a play of the game: if the run of $\mathcal{A}$ he constructs is accepting, and if the run the spoiler constructs on $\mathcal{B}$ is rejecting. The duplicator wins this game if he has a winning strategy, i.e., a recipe to move his pebble that guarantees that he wins. Such a winning strategy is "good-for-games," as it can only rely on the past. It can be used to transform winning strategies of $\mathcal{B}$, so that, if they were witnessing a good for games property or were good for an MDP, then the resulting strategy for $\mathcal{A}$ has the same property.

**Lemma 1 (Simulation Properties).** *For $\omega$-automata $\mathcal{A}$ and $\mathcal{B}$ the following holds.*

1. *If $\mathcal{A}$ simulates $\mathcal{B}$ then $\mathcal{L}(\mathcal{A}) \supseteq \mathcal{L}(\mathcal{B})$.*
2. *If $\mathcal{A}$ simulates $\mathcal{B}$ and $\mathcal{L}(\mathcal{A}) \subseteq \mathcal{L}(\mathcal{B})$ then $\mathcal{L}(\mathcal{A}) = \mathcal{L}(\mathcal{B})$.*
3. *If $\mathcal{A}$ simulates $\mathcal{B}$, $\mathcal{L}(\mathcal{A}) = \mathcal{L}(\mathcal{B})$, and $\mathcal{B}$ is GFG, then $\mathcal{A}$ is GFG.*
4. *If $\mathcal{A}$ simulates $\mathcal{B}$, $\mathcal{L}(\mathcal{A}) = \mathcal{L}(\mathcal{B})$, and $\mathcal{B}$ is GFM, then $\mathcal{A}$ is GFM.*

*Proof.* Facts (1) and (2) are well known observations. Fact (1) holds because an accepting run of $\mathcal{B}$ on a word $\alpha$ can be translated into an accepting run of $\mathcal{A}$ on $\alpha$ by using the winning strategy of $\mathcal{A}$ in the simulation game. Fact (2) follows immediately from Fact (1). Facts (3) and (4) follow by simulating the behaviour of $\mathcal{B}$ on each run. □

This observation allows us to use a family of state-space reduction techniques, in particular those based on language preserving translations for Büchi automata based on simulation relation [7,31,10,9]. This requires stronger notions of simulations, like direct and delayed simulation [9]. For the deterministic part of an LDBA, one can also use space reduction techniques for DBAs like [25].

**Corollary 1.** *All statespace reduction techniques that turn an NBA $\mathcal{A}$ into an NBA $\mathcal{B}$ that simulates $\mathcal{A}$ preserve GFG and GFM: if $\mathcal{A}$ is GFG or GFM, then $\mathcal{B}$ is GFG or GFM, respectively.*

#### 3.2 Constructing Slim GFM Automata

Let us fix Büchi automaton $\mathcal{B} = \langle \Sigma, Q, Q_0, \Delta, \Gamma \rangle$. We can write $\Delta$ as a function $\hat{\delta}\colon Q \times \Sigma \to 2^Q$ with $\hat{\delta}\colon (q,\sigma) \mapsto \{q' \in Q \mid (q,\sigma,q') \in \Delta\}$, which can be lifted to sets, using the deterministic transition function $\delta\colon 2^Q \times \Sigma \to 2^Q$ with $\delta\colon (S,\sigma) \mapsto \bigcup_{q\in S} \hat{\delta}(q,\sigma)$. We also define an operator, ndet, that translates deterministic transition functions $\delta\colon R \times \Sigma \to R$ to relations, using

$$\mathsf{ndet}\colon (R \times \Sigma \to R) \to 2^{R\times\Sigma\times R} \quad \text{with} \quad \mathsf{ndet}\colon \delta \mapsto \big\{(q,\sigma,q') \mid q' \in \delta(\{q\},\sigma)\big\}.$$

This is just an easy means to move back and forth between functions and relations, and helps one to visualize the maximal number of successors. We next define the variations of subset and breakpoint constructions that are used to define the well-known limit deterministic GFM automata—which we use in our proofs—and the slim GFM automata we construct. Let $3^Q := \{(S,S') \mid S' \subsetneq S \subseteq Q\}$ and $3^Q_+ := \{(S,S') \mid S' \subseteq S \subseteq Q\}$. We define the subset notation for the transitions and accepting transitions as $\delta_S, \gamma_S \colon 2^Q \times \Sigma \to 2^Q$ with

$$\delta_S \colon (S,\sigma) \mapsto \{q' \in Q \mid \exists q \in S.\ (q,\sigma,q') \in \Delta\} \text{ and}$$
$$\gamma_S \colon (S,\sigma) \mapsto \{q' \in Q \mid \exists q \in S.\ (q,\sigma,q') \in \Gamma\}.$$

We define the raw breakpoint transitions $\delta_R \colon 3^Q \times \Sigma \to 3^Q_+$ as $((S,S'),\sigma) \mapsto (\delta_S(S,\sigma), \delta_S(S',\sigma) \cup \gamma_S(S,\sigma))$. In this construction, we follow the set of reachable states (first set) and the states that are reachable while passing at least one of the accepting transitions (second set). To turn this into a breakpoint automaton, we reset the second set to the empty set when it equals the first; the transitions where we reset the second set are exactly the accepting ones. The breakpoint automaton $\mathcal{D} = \langle \Sigma, 3^Q, (Q_0,\emptyset), \delta_B, \gamma_B \rangle$ is defined such that, when $\delta_R \colon ((S,S'),\sigma) \mapsto (R,R')$, then there are three cases:

1. if $R = \emptyset$, then $\delta_B((S,S'))$ is undefined (or, if a complete automaton is preferred, maps to a rejecting sink),
2. else, if $R \neq R'$, then $\delta_B \colon ((S,S'),\sigma) \mapsto (R,R')$ is a non-accepting transition,
3. otherwise $\delta_B, \gamma_B \colon ((S,S'),\sigma) \mapsto (R',\emptyset)$ is an accepting transition.

Finally, we define transitions $\Delta_{SB} \subseteq 2^Q \times \Sigma \times 3^Q$ that lead from a subset to a breakpoint construction, and $\gamma_{2,1} \colon 3^Q \times \Sigma \to 3^Q$ that promote the second set of a breakpoint construction to the first set as follows.

1. $\Delta_{SB} = \Big\{(S,\sigma,(S',\emptyset)) \mid \emptyset \neq S' \subseteq \delta_S(S,\sigma)\Big\}$ are non-accepting transitions,
2. if $\delta_S(S',\sigma) = \gamma_S(S,\sigma) = \emptyset$, then $\gamma_{2,1}((S,S'),\sigma)$ is undefined, and
3. otherwise $\gamma_{2,1} \colon ((S,S'),\sigma) \mapsto (\delta_S(S',\sigma) \cup \gamma_S(S,\sigma), \emptyset)$ is an accepting transition.

We can now define standard limit deterministic good for MDP automata.

**Theorem 1.** *[11] $\mathcal{A} = \langle \Sigma, 2^Q \cup 3^Q, Q_0, \mathsf{ndet}(\delta_S) \cup \Delta_{SB} \cup \mathsf{ndet}(\delta_B), \mathsf{ndet}(\gamma_B) \rangle$ recognizes the same language as $\mathcal{B}$. It is limit deterministic and good for MDPs.*

We now show how to construct a slim GFM Büchi automaton.

**Theorem 2 (Slim GFM Büchi Automaton).** *The automaton*

$$\mathcal{S} = \langle \Sigma, 3^Q, (Q_0,\emptyset), \mathsf{ndet}(\delta_B) \cup \mathsf{ndet}(\gamma_{2,1}), \mathsf{ndet}(\gamma_B) \cup \mathsf{ndet}(\gamma_{2,1}) \rangle$$

*simulates $\mathcal{A}$. $\mathcal{S}$ is slim, language equivalent to $\mathcal{B}$, and good for MDPs.*

*Proof.* $\mathcal{S}$ is slim: its set of transitions is the union of two sets of deterministic transitions. We show that $\mathcal{S}$ simulates $\mathcal{A}$ by defining a strategy in the simulation game, which ensures that, if the spoiler produces a run $S_0 \ldots S_{j-1}(S_j,S'_j)(S_{j+1},S'_{j+1})\ldots$ for $\mathcal{A}$, then the duplicator produces a run $(T_0, T_0') \dots (T_{j-1}, T_{j-1}')(T_j, T_j')(T_{j+1}, T_{j-1}') \dots$ for $\mathcal{S}$, such that (1) $S_i \subseteq T_i$ holds for all $i \in \omega$, and (2) if there are two accepting transitions $\big((S_{k-1}, S_{k-1}'), \sigma_k, (S_k, S_k')\big)$ and $\big((S_{l-1}, S_{l-1}'), \sigma_l, (S_l, S_l')\big)$ with $k < l$, there is an $k < m \leq l$, such that $\big((T_{m-1}, T_{m-1}'), \sigma_m(T_m, T_m')\big)$ is accepting.

To obtain this, we describe a winning strategy for the duplicator while arguing inductively that it mainains (1). Note that (1) holds initially ($T_0 = S_0$, induction basis).

**Initial Phase:** Every move of the spoiler—with some letter $\sigma$—that uses a transition from $\delta_S$—the subset part of $\mathcal{A}$—is followed by a move from $\delta_B$ with the same letter $\sigma$. When the duplicator follows this strategy the following holds: when, after a pair of moves, the pebble of the spoiler is on state $S \subseteq Q$, then the pebble of the duplicator is on some state $(S, S')$. In particular, (1) is preserved during this phase (induction step).

**Transition Phase:** The one spoiler move—with some letter $\sigma$—that uses a transition from $\Delta_{SB}$—the transition to the breakpoint part of $\mathcal{A}$—is followed by a move from $\delta_B$ with the same letter $\sigma$. When the duplicator follows this strategy, and when, after the pair of moves, the pebble of the spoiler is on state $(S, \emptyset)$, then the pebble of the duplicator is on some state $(T, T')$ with $S \subseteq T$. In particular, (1) is preserved (induction step).

**Final Phase:** When the spoiler moves from some state $(S, S')$—with some letter $\sigma$—that uses a transition from $\delta_B$—the breakpoint part of $\mathcal{A}$—to $(\bar{S}, \bar{S}')$, and when the duplicator is in some state $(T, T')$, then the duplicator does the following. He calculates $(\bar{T}, \emptyset) = \gamma_{2,1}\big((T, T'), \sigma\big)$ and checks if $\bar{S} \subseteq \bar{T}$ holds. If $\bar{S} \subseteq \bar{T}$ holds, he plays this transition from $\gamma_{2,1}$ (with the same letter $\sigma$). Otherwise, he plays the transition from $\delta_B$ (with the same letter $\sigma$). In either case (1) is preserved (induction step), which closes the inductive argument for (1).

Note that no accepting transition of $\mathcal{A}$ is passed in the initial or tansition phase, so the two accepting transitions from (2) must both fall into the final phase.

To show (2), we first observe that $S_k' = \emptyset$, and thus $S_k' \subseteq T_k'$ holds. Assuming for contradition that all transitions of $\mathcal{S}$ for $\sigma_{k+1} \dots \sigma_{l-1}$ are non-accepting, we obtain—using (1)—by a straightforward inductive argument that $S_i' \subseteq T_i'$ for all $i$ with $k \leq i < l$. (Note that transitions in $\delta_B$ are accepting when they are also be in $\gamma_B$.)

Using that $S_l = \delta_S(S_{l-1}', \sigma_l) \cup \gamma_S(S_{l-1}, \sigma_l) \subseteq \delta_S(T_{l-1}', \sigma_l) \cup \gamma_S(T_{l-1}, \sigma_l)$ holds, the spoiler uses an accepting transition from $\gamma_{2,1}$ in this step.

Using Lemma 1, it now suffices to show that the language of $\mathcal{S}$ is included in the language of $\mathcal{B}$. To show this, we simply argue that an accepting run $\rho = (Q_0, Q_0'), (Q_1, Q_1'), (Q_2, Q_2'), (Q_3, Q_3'), \dots$ of $\mathcal{S}$ on an input word $\alpha = \sigma_0, \sigma_1, \sigma_2, \dots$ can be interpreted as a forest of finitely many finitely branching trees of overall infinite size, where all infinite branches are accepting runs of $\mathcal{B}$. Kőnig's Lemma then proves the existence of an accepting run of $\mathcal{B}$.

This forest is the usual one. The nodes are labeled by states of $\mathcal{B}$, and the roots (level 0) are the initial states of $\mathcal{B}$. Let $I = \big\{i \in \mathbb{N} \mid \big((Q_{i-1}, Q_{i-1}'), \sigma_{i-1}, (Q_i, Q_i')\big) \in \Gamma := \mathsf{ndet}(\gamma_B) \cup \mathsf{ndet}(\gamma_{2,1})\big\}$ be the set of positions after accepting transitions in $\rho$. We define the predecessor function $\mathsf{pred}\colon \mathbb{N} \to I \cup \{0\}$ with $\mathsf{pred}\colon i \mapsto \max\big\{j \in I \cup \{0\} \mid j < i\big\}$.

We call a node with label $q_l$ on level $l$ an end-point if one of the following applies: (1) $q_l \notin Q_l$ or (2) $l \in I$ and for all $j$ such that $\mathsf{pred}(l) \leq j < l$, where $q_j$ is the label of the ancestor of this node on level $j$, we have $(q_j, \sigma_j, q_{j+1}) \notin \Gamma$.

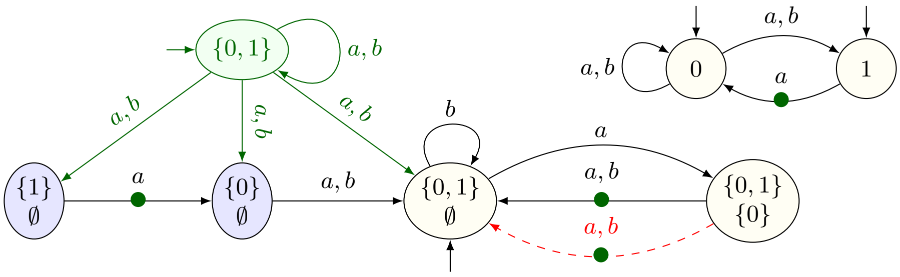

**Fig. 2.** An NBA for $\mathsf{G}\,\mathsf{F}\,a$ (in the upper right corner) together with an SLDBA and a slim NBA constructed from it. The SLDBA and the slim NBA are shown sharing their common part. State $\{0,1\}$, produced by the subset construction, is the initial state of the SLDBA, while state $(\{0,1\},\emptyset)$—the initial state of the breakpoint construction—is the initial state of the slim NBA. States $(\{1\},\emptyset)$ and $(\{0\},\emptyset)$ are states of the breakpoint construction that only belong to the SLDBA because they are not reachable from $(\{0,1\},\emptyset)$. The transitions out of $\{0,1\}$, except the self loop, belong to $\Delta_{SB}$. The dashed-line transition from $(\{0,1\},\{0\})$ belongs to $\gamma_{2,1}$

(1) may only happen after a transition from $\gamma_{2,1}$ has been taken, and the $q_l$ is not among the states that is traced henceforth. (2) identifies parts of the run tree that do not contain an accepting transition.

A node labeled with $q_l$ on level $l$ that is not an endpoint has $\left|\delta_S(q_l,\sigma_l)\right|$ children, labeled with the different elements of $\delta_S(q_l,\sigma_l)$. It is now easy to show by induction over $i$ that the following holds.

1. For all $q \in Q_i$, there is a node on level $i$ labeled with $q$.
2. For $i \notin I$ and $q \in Q'_i$, there is a node labeled $q$ on level $i$, a $j$ with $\mathsf{pred}(i) \leq j < i$, and ancestors on level $j$ and $j+1$ labeled $q_j$ and $q_{j+1}$, such that $(q_j,\sigma_j,q_{j+1}) \in \Gamma$. (The 'ancestor' on level $j+1$ might be the state itself.)
   For $i \in I$ and $q \in Q'_i$, there is a node labeled $q$ on level $i$, which is not an end point.

Consequently, the forest is infinite, finitely branching, and finitely rooted, and thus contains an infinite path. By construction, this path is an accepting run of $\mathcal{B}$. □

The resulting automata are simple in structure and enable symbolic implementation (See Fig. 2). It cannot be expected that there are much smaller good for MDP automata, as its explicit construction is the only non-polynomial part in model checking MDPs.

**Theorem 3.** *Constructing a GFM Büchi automaton $G$ that recognizes the models of an LTL formula $\varphi$ requires time doubly exponential in $\varphi$, and constructing a GFM Büchi automaton $G$ that recognizes the language of an NBA $\mathcal{B}$ requires time exponential in $\mathcal{B}$.*

*Proof.* As resulting automata are GFM, they can be used to model check MDPs $\mathcal{M}$ against this property, with cost polynomial in product of $\mathcal{M}$ and $\mathcal{G}$. If $\mathcal{G}$ could be produced faster (and if they could, consequently be smaller) than claimed, it will contradict the 2-ExpTime- and ExpTime-hardness [4] of these model checking problems. □

### 4 Accepting End-Component Simulation

An *end-component* [5,2] of an MDP $\mathcal{M}$ is a sub-MDP $\mathcal{M}'$ of $\mathcal{M}$ such that its underlying graph is strongly connected. A *maximal* end-component is maximal under set-inclusion. Every state of an MDP belongs to at most one maximal end-component.

**Theorem 4 (End-Component Properties. Theorem 3.1 and Theorem 4.2 of [5]).** *Once an end-component $C$ of an MDP is entered, there is a strategy that visits every state-action combination in $C$ infinitely often with probability* 1 *and stays in $C$ forever.*

*For a product MDP, an* accepting end-component *(AEC) is an end-component that contains some transition in $\Gamma^\times$. There is a positional pure strategy for an AEC $C$ that surely stays in $C$ and almost surely visits a transition in $\Gamma^\times$ infinitely often.*

*For a product MDP, there is a set of disjoint accepting end-components such that, from every state, the maximal probability to reach the union of these accepting end-components is the same as the maximal probability to satisfy $\Gamma^\times$. Moreover, this probability can be realized by combining a positional pure (reachability) strategy outside of this union with the aforementioned positional pure strategies for the individual AECs.*

Lemma 1 shows that the GFM property is preserved by simulation: For language-equivalent automata $\mathcal{A}$ and $\mathcal{B}$, if $\mathcal{A}$ simulates $\mathcal{B}$ and $\mathcal{B}$ is GFM, then $\mathcal{A}$ is also GFM. However, a GFM automaton may not simulate a language-equivalent GFM automaton. (See Figure 3.) Therefore we introduce a coarser preorder, Accepting End-Component (AEC) simulation, that exploits the finiteness of the MDP $\mathcal{M}$. We rely on Theorem 4 to focus on positional pure strategies for $\mathcal{M} \times \mathcal{B}$. Under such strategies, $\mathcal{M} \times \mathcal{B}$ becomes a Markov chain [2] such that almost all its runs have the following properties:

- They will eventually reach a leaf strongly connected component (LSCC) in the Markov chain.
- If they have reached a LSCC $L$, then, for all $\ell \in \mathbb{N}$, all sequences of transitions of length $\ell$ in $L$ occur infinitely often, and no other sequence of length $\ell$ occurs.

With this in mind, we can intuitively ask the spoiler to pick a run through this Markov chain, and to disclose information about this run. Specifically, we can ask her to signal when she has reached an accepting LSCC[5] in the Markov chain, and to provide information about this LSCC, in particular information entailed by the full list of sequences of transitions of some fixed length $\ell$ described above. Runs that can be identified to either not reach an accepting LSCC, to visit transitions not in this list, or to visit only a subset of sequences from this list, form a 0 set. In the simulation game we define below, we make use of this observation to discard such runs.

A simulation game can only use the syntactic material of the automata—-neither the MDP nor the strategy are available. The information the spoiler may provide cannot explicitly refer to them. What the spoiler may be asked to provide is information on when she has entered an accepting LSCC, and, once she has signaled this, which sequences of length $l$ of *automata* transitions of $\mathcal{B}$ occur in the LSCC. The sequences of automata transitions are simply the projections on the automata transitions from the

[5] There is nothing to show when a non-accepting LSCC is reached—if $\mathcal{B}$ rejects, then $\mathcal{A}$ may reject too—nor when no LSCC is reached, as this occurs with probability 0.

sequences of transitions of length $\ell$ that occur in the LSCC $L$. We call this information a *gold-brim accepting end-component claim* of length $\ell$, $\ell$-GAEC claim for short.

The term "gold-brim" in the definition indicates that this is a powerful approach, but not one that can be implemented efficiently. We will define weaker, efficiently implementable notions of accepting end-component claims (AEC claims) later.

The AEC simulation game is very similar to the simulation game of Section 3.1. Both players produce an infinite run of their respective automata. If the spoiler makes an AEC claim, e.g., an $\ell$-GAEC claim, we say that her run *complies* with it if, starting with the transition when the AEC claim is made, all states, transitions, or sequences of transitions in the claim appear infinitely often, and all states, transitions, and sequences of transitions the claim excludes do not appear. For an $\ell$-GAEC claim, this means that all of the sequences of transitions of length $\ell$ in the claim occur infinitely often, and no other sequence of length $\ell$ occurs henceforth.

Thus, like a classic simulation game, an $\ell$-GAEC simulation game is started by the spoiler, who places her pebble on an initial state of $\mathcal{B}$. Next, the duplicator puts his pebble on an initial state of $\mathcal{A}$. The two players then take turns, always starting with the spoiler choosing an input letter and an according transition from $\mathcal{B}$, followed by the duplicator choosing a transition for the same letter in $\mathcal{A}$.

Different from the classic simulation game, in an $\ell$-GAEC simulation game, the spoiler has an additional move that she can (and, in order to win, has to) perform once in the game: In addition to choosing a letter and a transition, she can claim that she has reached an accepting end-component, and provide a complete list of sequences of automata transitions of length $\ell$ that can henceforth occur. This store is maintained, and never updated. It has no further effect on the rules of the game: Both players produce an infinite run of their respective automata. The duplicator has four ways to win:

1. if the spoiler never makes an AEC claim,
2. if the run of $\mathcal{A}$ he constructs is accepting,
3. if the run the spoiler constructs on $\mathcal{B}$ does not comply with the AEC claim, and
4. if the run that the spoiler produces is not accepting.

For $\ell$-GAEC claims, (4) simply means that the set of transitions defined by the sequences does not satisfy the Büchi, parity, or Rabin acceptance condition.

**Theorem 5.** *[$\ell$-GAEC Simulation] If $\mathcal{A}$ and $\mathcal{B}$ are language equivalent automata, $\mathcal{B}$ is GFM, and there exists an $\ell$ such that $\mathcal{A}$ $\ell$-GAEC simulates $\mathcal{B}$, then $\mathcal{A}$ is GFM.*

For the proof, we use an arbitrary (but fixed) MDP $\mathcal{M}$, and an arbitrary (but fixed) pure optimal positional strategy $\mu$ for $\mathcal{M} \times \mathcal{B}$, resulting in the Markov chain $(\mathcal{M} \times \mathcal{B})_\mu$. We assume w.l.o.g. that the accepting LSCCs in $(\mathcal{M} \times \mathcal{B})_\mu$ are identified, e.g., by a bit.

Let $\tau$ be a winning strategy of the duplicator in an $\ell$-GAEC simulation game. Abusing notation, we let $\tau \circ \mu$ denote the finite-memory strategy[6] obtained from $\mu$ and $\tau$ for $\mathcal{M} \times \mathcal{A}$, where $\tau$ is acting only on the automata part of $(\mathcal{M} \times \mathcal{B})$, and where the spoiler

[6] The strategy $\tau$ consists of one sub-strategy to be used before the AEC claim is made and one sub-strategy for each possible $\ell$-GAEC claim. The memory of $\tau \circ \mu$ tracks the position in $(\mathcal{M} \times \mathcal{B})_\mu$. When an accepting LSCC is detected (via the marker bit) analysis of $(\mathcal{M} \times \mathcal{B})_\mu$ reveals the only possible $\ell$-GAEC claim. This claim is used to select the right entry from $\tau$.

makes the move to the end-component when she is in some LSCC $B$ of $(\mathcal{M} \times \mathcal{B})_\mu$ and gives the full list of sequences of transitions of length $\ell$ that occur in $B$.

*Proof.* As $\mathcal{B}$ is good for MDPs, we only have to show that the chance of winning in $(\mathcal{M} \times \mathcal{A})_{\tau \circ \mu}$ is at least the chance of winning in $(\mathcal{M} \times \mathcal{B})_\mu$. The chance of winning in $(\mathcal{M} \times \mathcal{B})_\mu$ is the chance of reaching an accepting LSCC in $(\mathcal{M} \times \mathcal{B})_\mu$. It is also the chance of reaching an accepting LSCC $L \in (\mathcal{M} \times \mathcal{B})_\mu$ *and*, after reaching $L$, to see exactly the sequences of transitions of length $\ell$ that occur in $L$, and to see all of them infinitely often.

By construction, $\tau \circ \mu$ will translate those runs into accepting runs of $(\mathcal{M} \times \mathcal{A})_{\tau \circ \mu}$, such that the chance of an accepting run of $(\mathcal{M} \times \mathcal{A})_{\tau \circ \mu}$ is at least the chance of an accepting run of $(\mathcal{M} \times \mathcal{B})_\mu$. As $\mu$ is optimal, the chance of winning in $\mathcal{M} \times \mathcal{A}$ is at least the chance of winning in $\mathcal{M} \times \mathcal{B}$. As $\mathcal{B}$ is GFM, this is the chance of $\mathcal{M}$ producing a run accepted by $\mathcal{B}$ (and thus $\mathcal{A}$) when controlled optimally, which is an upper bound on the chance of winning in $\mathcal{M} \times \mathcal{A}$. □

An $\ell$-GAEC simulation, especially for large $\ell$, results in very large state spaces, because the spoiler has to list all sequences of transitions of $\mathcal{B}$ of length $\ell$ that will appear infinitely often. No other sequence of length $\ell$ may then appear in the run[7]. This can, of course, be prohibitively expensive.

As a compromise, one can use coarser-grained information at the cost of reducing the duplicator's ability of winning the game. E.g., the spoiler could be asked to only reveal a transition that is repeated infinitely often, plus (when using more powerful acceptance conditions than Büchi), some acceptance information, say the dominating priority in a parity game or a winning Rabin pair. This type of coarse-grained claim can be refined slightly by allowing the *duplicator* to change at any time the transition that is to appear infinitely often to the transition just used by the spoiler. Generally, we say that an AEC simulation game is any simulation game, where

- the spoiler provides a list of states, transitions, or sequences of transitions that will occur infinitely often and a list of states, transitions, or sequences of transitions that will not occur in the future when making her AEC claim, and
- the duplicator may be able to update this list based on his observations,
- there exists some $\ell$-GAEC simulation game such that a winning strategy of the spoiler translates into a winning strategy of the spoiler in the AEC simulation game.

The requirement that a winning spoiler strategy translates into a winning spoiler strategy in an $\ell$-GAEC game entails that AEC simulation games can prove the GFM property.

**Corollary 2.** *[AEC Simulation] If $\mathcal{A}$ and $\mathcal{B}$ are language equivalent automata, $\mathcal{B}$ is good for MDPs, and $\mathcal{A}$ AEC-simulates $\mathcal{B}$, then $\mathcal{A}$ is good for MDPs.*

[7] The AEC claim provides information about the accepting LSCC in the product under the chosen pure positional strategy. When the AEC claim requires the exclusion of states, transitions, or sequences of transitions, then they are therefore surely excluded, whereas when it requires inclusion of, and thus inclusion of infinitely many occurrances of, states, trasitions, or sequences of transitions, then they (only) occur almost surely infinitely often. Yet, runs that do not contain them all infinitely often form a zero set, and can thus be ignored.

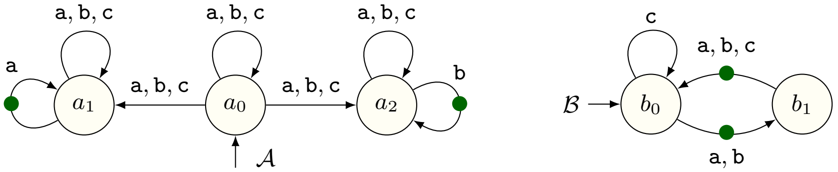

**Fig. 3.** Automata $\mathcal{A}$ (left) and $\mathcal{B}$ (right) for $\varphi = (\mathsf{G}\,\mathsf{F}\,\mathtt{a}) \vee (\mathsf{G}\,\mathsf{F}\,\mathtt{b})$. The dotted transitions are accepting. The NBA $\mathcal{A}$ does not simulate the DBA $\mathcal{B}$: $\mathcal{B}$ can play $a$'s until $\mathcal{A}$ moves to either the state on the left, or the state on the right. $\mathcal{B}$ then wins by henceforth playing only $b$'s or only $a$'s. However, $\mathcal{A}$ is good for MDPs. It wins the AEC simulation game by waiting until an AEC is reached (by $\mathcal{B}$), and then check if $a$ or $b$ occurs infinitely often in this AEC. Based on this knowledge, $\mathcal{A}$ can make its decision. This can be shown by AEC simulation if $\mathcal{B}$ has to provide sufficient information, such as a list of transitions—or even a list of letters—that occur infinitely often. The amount of information the spoiler has to provide determines the strength of the AEC simulation used. If, e.g., $\mathcal{B}$ only has to reveal one accepting transition of the end-component, then it can select an end-component where the revealed transition is $(b_1, c, b_0)$, which does not provide sufficient information. Whereas, if the duplicator is allowed to update the transition, then the duplicator wins by updating the recorded transition to the next $a$ or $b$ transition

Of course, for every AEC simulation, one first has to prove that winning strategies for the spoiler translate. We have used two simple variations of the AEC simulation games:

**accepting transition:** the spoiler may only make her AEC claim when taking an accepting transition; this transition—and no other information—is stored, and the spoiler commits to—and commits only to—seeing this transition infinitely often;

**accepting transition with update:** different to the *accepting transition* AEC simulation game, the duplicator can—but does not have to—update the stored accepting transition whenever the spoiler passes by an accepting transition.

**Theorem 6.** *Both, the* accepted transition *and the* accepted transition with update *AEC simulation, can be used to establish the good for MDPs property.*

To show this, we describe the strategy translations in accordance with Corollary 2.

*Proof.* In both cases, the translation of a winning strategy of the spoiler for the 1-GAEC simulation game are straightforward: The spoiler essentially follows her winning strategy from the 1-GAEC simulation game, with the extra rule that she will make her AEC claim to the duplicator on the first accepting transition on or after her AEC claim in the 1-GAEC claim. If the duplicator is allowed to update the transition, this information is ignored by the spoiler—she plays according to her winning strategy from the 1-GAEC simulation game. Naturally, the resulting play will comply with her 1-GAEC claim, and will thus also be winning for the—weaker—AEC claim made to the duplicator. □

We use AEC simulation to identify GFM automata among the automata produced (e.g., by SPOT [8]) at the beginning of the transformation. Figure 3 shows an example for which the duplicator wins the AEC simulation game, but loses the ordinary simulation game. Candidates for automata to simulate are, e.g., the slim GFM Büchi automata and the limit deterministic Büchi automata discussed above.

## 5 Evaluation

### 5.1 Size of General Büchi Automata for Probabilistic Model Checking

As discussed, automata that simulate slim automata or SLDBAs are good for MDPs. This fact can be used to allow Büchi automata produced from general-purpose tools such as SPOT's [8] ltl2tgba rather than using specialized automata types. Automata produced by such tools are often smaller because such general-purpose tools are highly optimized and not restricted to producing slim or limit deterministic automata. Thus, one produces an arbitrary Büchi automaton using any available method, then transforms this automaton into a slim or limit deterministic automaton, and finally checks whether the original automaton simulates the generated one.

We have evaluated this idea on random LTL formulas produced by SPOT's tool randltl. We have set the tree size, which influences the size of the formulas, to 50, and have produced 1000 formulas with 4 atomic propositions each. We left the other values to their defaults. We have then used SPOT's ltl2tgba (version 2.7) to turn these formulas into non-generalized Büchi automata using default options. Finally, for each automaton, we have used our tool to check whether the automaton simulates a limit deterministic automaton that we produce from this automaton. For comparison, we have also used Owl's [29] tool ltl2ldba (version 19.06.03) to compute limit deterministic non-generalized Buchi automata. We have also used the option of this tool to compute Büchi automata with a nondeterministic initial part. We used 10 minute timeouts.

Of these 1000 formulas, 315 can be transformed to deterministic Büchi automata. For an additional 103 other automata generated, standard simulation sufficed to show that they are GFM. For a further 11 of them, the simplest AEC simulation (the spoiler chooses an accepting transition to occur infinitely often) sufficed, and another 1 could be classed GFM by allowing the duplicator to update the transition. 501 automata turned out to be nonsimulatable and for 69 we did not get a decision due to a timeout.

For the LTL formulas for which ltl2tgba could not produce deterministic automata, but for which simulation could be shown, the number of states in the generated automata was often lower than the number of states in the automata produced by Owl's tools. On average, the number of states per automaton was ≈15.21 for SPOT's ltl2tgba; while for Owl's ltl2ldba it was ≈46.35. The extended version of this paper [13] contains more details about the evaluation.

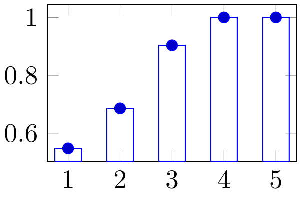

**Fig. 4.** Deciles ratio ltl2tgba /semi-deterministic automata

Let us consider the ratio between the size of automata produced by ltl2tgba and the size of semi-deterministic automata produced by Owl. The average of this number for all automata that are not deterministic and that can be simulated in some way is $\approx 1.0335$. This means that on average, for these automata, the semi-deterministic automata are slightly smaller. If we take a look at the first 5 deciles depicted in Fig. 4, we see that there is a large number of formulas for which ltl2tgba and Owl produce automata of the same size. For around 24.3478% of the cases, automata by SPOT are smaller than those produced by Owl (ratio $< 1$).

#### 5.2 GFM Automata and Reinforcement Learning

SLDBAs have been used in [12] for model-free reinforcement learning of $\omega$-regular objectives. While the Büchi acceptance condition allows for a faithful translation of the objective to a scalar reward, the agent has to learn how to control the automaton's nondeterministic choices; that is, the agent has to learn when the SLDBA should cross from the initial component to the accepting component to produce a successful run of a behavior that satisfies the given objective.

Any GFM automaton with a Büchi acceptance condition can be used instead of an SLDBA in the approach of [12]. While in many cases SLDBAs work well, GFM automata that are not limit-deterministic may provide a significant advantage.

Early during training, the agent relies on uniform random choices to discover policies that lead to successful episodes. This includes randomly resolving the automaton nondeterminism. If random choices are unlikely to produce successful runs of the automaton in case of behaviors that should be accepted, learning is hampered because good behaviors are not rewarded. Therefore, GFM automata that are more likely to accept under random choices will result in the agent learning more quickly. We have found the following properties of GFM automata to affect the agent's learning ability.

**Low branching degree.** A low branching degree presents the agent with fewer alternatives, reducing the expected number of trials before the agent finds a good combination of choices. Consider an MDP and an automaton that require a specific sequence of $k$ nondeterministic choices in order for the automaton to accept. If at each choice there are $b$ equiprobable options, the correct sequence is obtained with probability $b^{-k}$.

**Cautiousness.** An automaton that enables fewer nondeterministic choices for the same finite input word gives the agent fewer chances to choose wrong. The slim automata construction has the interesting property of "collecting hints of acceptance" before a nondeterministic choice is enabled because $S'$ has to be nonempty for a $\gamma_{2,1}$ transition to be present and that requires going through at least one accepting transition.

**Forgiveness.** Mistakes made in resolving nondeterminism may be irrecoverable. This is often true of SLDBAs meant for model checking, in which jumps are made to select a subformula to be eventually satisfied. However, general GFM automata, thanks also to their less constrained structure, may be constructed to "forgive mistakes" by giving more chances of picking a successful run.

Figure 5 compares a typical SLDBA to an automaton that is not limit-deterministic and is not produced by the breakpoint construction, but is proved GFM by AEC simulation. This latter automaton has a nondeterministic choice in state $q_0$ on letter $x \wedge \neg y$ that can be made an unbounded number of times. The agent may choose $q_1$ repeatedly even if eventually $\mathsf{F}\,\mathsf{G}\,x$ is false and $\mathsf{G}\,\mathsf{F}\,y$ is true. With the SLDBA, on the other hand, there is no room for error.

**A Case Study.** We compared the effectiveness in learning to control a cart-pole model of three automata for the property $\big((\mathsf{F}\,\mathsf{G}\,x) \vee (\mathsf{G}\,\mathsf{F}\,y)\big) \wedge \mathsf{G}\,\mathtt{safe}$. The safety component of the objective is to keep the pole balanced and the cart on the track. The left two thirds of the track alternate between $x$ and $y$ at each step. The right third is always labeled $y$, but in order to reach it, the cart has to cross a barrier, with probability $1/3$ of failing.

The three automata are an SLDBA (4 states), a slim automaton (8 states), and a handcrafted forgiving automaton (4 states) similar to the one of Fig. 5.

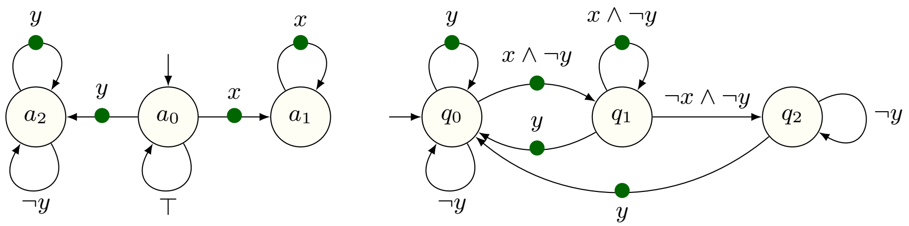

**Fig. 5.** Two GFM automata for $(\mathsf{F}\,\mathsf{G}\,x) \vee (\mathsf{G}\,\mathsf{F}\,y)$. SLDBA (left), and forgiving (right)

Training of the continuous-statespace model employed PPO [28] as implemented in OpenAI Baselines [6]. Figure 6 shows the learning curves for the three automata averaged over ten runs. They underline the importance of choosing the right automaton in RL. Training parameters, more details on the model, and additional examples can be found in the extended version of this paper [13].

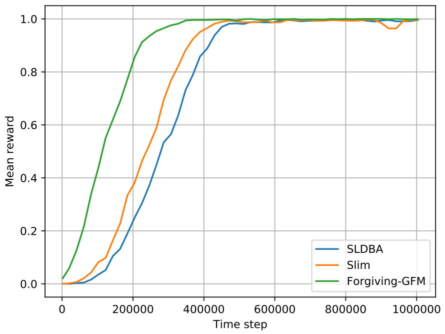

**Fig. 6.** Learning curves

### 6 Conclusion

We have defined the class of automata that are *good for MDPs*—nondeterministic automata that can be used for the analysis of MDPs—and shown it to be closed under different simulation relations. This has multiple favorable implications for model checking and reinforcement learning. Closure under classic simulation opens a rich toolbox of statespace reduction techniques that come in handy to push the boundary of analysis techniques, while the more powerful (and more expensive) AEC simulation has promise to identify source automata that happen to be good for MDPs.

The wider class of GFM automata also shows promise: the slim automata we have defined to tame the branching degree while retaining the desirable Büchi condition for reinforcement learning are able to compete even against optimized SLDBAs.

As outlined in Section 5.2, a low branching degree, cautiousness, and forgiveness make automata particularly well-suited for learning. From a practical point of view, much of the power of this new approach is in harnessing the power of simulation for learning, and forgiveness is closely related to simulation.

The natural follow-up research is to tap the full potential of simulation-based statespace reduction instead of the limited version that we have implemented. Besides using this to get the statespace small—useful for model checking—we will use simulation to construct forgiving automata, which is promising for reinforcement learning.

Datasets generated and analyzed during the current study are available at: https://doi.org/10.6084/m9.figshare.11882739 [35,36]
